# Benign thyroid disease and dietary factors in thyroid cancer: a case–control study in Kuwait

**DOI:** 10.1038/sj.bjc.6600303

**Published:** 2002-06-05

**Authors:** A Memon, A Varghese, A Suresh

**Affiliations:** Department of Community Medicine and Behavioural Sciences, Faculty of Medicine, Kuwait University, PO Box 24923, Safat 13110, Kuwait; Kuwait Cancer Control Centre, PO Box 42262, Shuwaikh 70563, Kuwait

**Keywords:** benign thyroid disease, dietary factors, thyroid cancer, aetiology, case–control study, Kuwait

## Abstract

We conducted a population-based study of 313 case–control pairs in Kuwait to examine the aetiology of thyroid cancer, the second most common neoplasm among women in this and several other countries in the Gulf region. Among the demographic variables, individuals with 12+ years of education had a significantly reduced risk of thyroid cancer (OR=0.6; 95% CI: 0.3–0.9). The average age at diagnosis (±s.d.) of thyroid cancer was 34.7±11 years in women and 39±13.4 years in men. History of thyroid nodule was reported only by cases (*n*=34; 10.9%; lower 95% CI: 12.0); and goitre by 21 cases and four controls (OR=5.3; 95% CI: 1.8–15.3). There was no significant increase in risk with history of hypothyroidism (OR=1.8) or hyperthyroidism (OR=1.7). For any benign thyroid disease, the OR was 6.4 (95% CI: 3.4–12.0); and the population attributable risk was about 26% (95% CI: 21.1–30.9). Stepwise regression analysis showed that high consumption of processed fish products (OR=2.2; 95% CI: 1.6–3.0) fresh fish (OR=0.5; 95% CI: 0.4–0.7) and chicken (OR=1.7; 95% CI: 1.2–2.3) were independently associated with thyroid cancer with significant dose-response relationships. Among the thyroid cancer patients who reported high consumption of fish products, a large majority also reported high consumption of fresh fish (98%) and shellfish (68%). No clear association emerged with consumption of cruciferous vegetables. These data support the hypothesis that hyperplastic thyroid disease is strongly related to thyroid cancer; and that habitual high consumption of various seafoods may be relevant to the aetiology of thyroid cancer. The association with chicken consumption requires further study.

*British Journal of Cancer* (2002) **86**, 1745–1750. doi:10.1038/sj.bjc.6600303 www.bjcancer.com

© 2002 Cancer Research UK

## 

In most countries, thyroid cancer accounts for approximately 1–5% of all cancers in females and <2% in males. The age-standardised incidence rates (per 100 000) of thyroid cancer, across most populations, vary from about 2–10 in females and 1–3 in males (Parkin *et al*, 1997). Since the late 1970s, thyroid cancer has consistently been the second most commonly recorded neoplasm (after breast) among Kuwaiti women. During the period 1994–1998, thyroid cancer accounted for 8.1 and 2.1% of all cancers among Kuwaiti women and men, respectively; and 8.7 and 3.3% of all cancers among non-Kuwaiti (expatriate) women and men, respectively. Similarly high relative frequency and incidence rates of the disease have also been observed in other countries in the Gulf region (Figure 1

**Figure 1:**
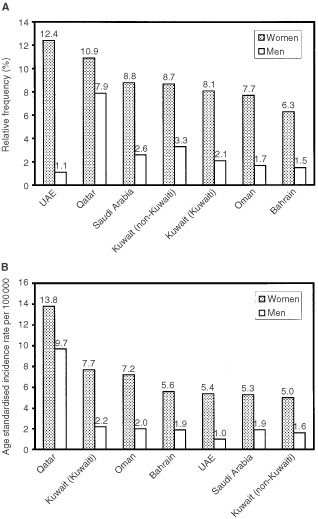
Relative frequency (**A**) and age standardised incidence rates (**B**) of thyroid cancer in the Gulf countries. Source: Kuwait Cancer Registry (1994–98); Gulf Centre for Cancer Registration, *Cancer incidence report* (Bahrain, Qatar, UAE, 1998); *Cancer incidence in Oman* (1997); *Cancer incidence report*, Saudi Arabia (1994–96).

).

Evidence from epidemiological studies suggests that exposure to ionising radiation, especially during childhood and adolescence, is the main risk factor for thyroid cancer (Ron *et al*, 1995); and history of hyperplastic thyroid disease (i.e., nodule/adenoma and goitre) is an important determinant of the cancer (Franceschi *et al*, 1999). The study of dietary habits and micro-nutrients in thyroid cancer has been prompted by the essential role of iodine in thyroid function and the potential influence of iodine-rich seafood and goitrogenic vegetables (World Cancer Research Fund, 1997). We conducted a population-based case–control study in Kuwait to examine the major aetiological hypotheses for thyroid cancer.

## SUBJECTS AND METHODS

The study population and methods are described in detail elsewhere (Memon *et al*, 2002). Briefly, our study was conducted at the Kuwait Cancer Control Centre (KCCC) which is the only specialised cancer treatment and follow-up hospital in the country. Due to the relatively high incidence of thyroid cancer in Kuwait, a special follow-up clinic is held every week at the KCCC for thyroid cancer patients. A population-based cancer registry (Kuwait Cancer Registry) has been established at the centre since 1979. We first collected information on all the 874 patients diagnosed with thyroid cancer during the period 1 January 1981 to 30 June 1999 from the records of the cancer registry and KCCC. Of these, 611 (69.9%) were females and 263 (30.1%) were males. Among these patients, 66 were known to have died (42 females, 24 males). In order to achieve sufficient statistical power for a meaningful analysis, we decided to include as many of the patients as possible who were diagnosed since 1 January 1981. This rationale was based on the fact that there were relatively small numbers of new cases each year, particularly among the Kuwaiti nationals, and that many non-Kuwaiti patients return to their home country after diagnosis or initial treatment. It is also noteworthy that thyroid cancer has one of the most favourable prognosis with all-stage relative 5-year survival rates of around 95% for women and 92% for men (Ron, 1996). Patients were considered eligible for inclusion in the study if thyroid cancer had occurred as the first primary cancer and if they were alive, aged ⩽70 years, and resident in Kuwait during the data collection period (1 May 1998–30 June 1999). All eligible patients who attended the weekly thyroid cancer clinic or other in- and outpatient departments at the KCCC during this period were solicited to participate in the study. Other eligible patients, who did not visit the KCCC during this period, were traced through the information available in medical records and where possible, were invited to visit the hospital for an interview. The study finally included a total of 313 patients with thyroid cancer (238 females, 75 males).

The district of residence was determined for each thyroid cancer patient and the local primary health care clinic was visited to select a suitably matched control subject. Kuwait has a distinctive network of primary care clinics in terms of its accessibility and the wide range of services offered at these clinics; and all residents in the district have an equal opportunity to visit the local clinic. One control subject was individually matched to each thyroid cancer patient, based on year of birth (within 3 years), gender, nationality, and district of residence. Subjects were considered eligible to serve as controls if they were visiting the primary care clinic for minor complaints, or accompanying such persons, or visiting for any other purpose. Of the 340 eligible control subjects, solicited for participation in the study, 27 refused to be interviewed, yielding a response rate of 92.1% (313 out of 340).

All cases and controls included in the study were initially contacted and interviewed in-person during the period 1 May 1998–30 June 1999. The data were collected by using a structured questionnaire which included information on: (i) sociodemographic characteristics; (ii) gynaecological and reproductive history; (iii) medical history (including autoimmune and chronic diseases and benign thyroid disease); (iv) family history (including thyroid disease and cancer); (v) habitual diet (frequency of consumption of 13 dietary items into one of the following six categories: never, occasional–few times a year, 1–3 times per month, 1 day per week, 2–4 days per week, or 5–7 days per week); and (vi) clinical and histopathological information (abstracted from the records of the cancer registry and KCCC).

### Analysis

For each control a ‘pseudo-diagnosis’ date was determined, the date on which the subject was the same age as his/her matching case was at the time of diagnosis. The analysis of hyperplastic thyroid disease was restricted to events that occurred ⩾3 years before the diagnosis (cases) or pseudo-diagnosis (controls) date. The analysis of data on smoking was based on smoking status at the time of diagnosis/pseudo-diagnosis. For dietary items, the intake categories were grouped into low (never or occasional–few times a year), moderate (1–3 times per month or 1 day per week), and high consumption (2–4 days per week or 5–7 days per week). We used conditional logistic regression method, as described by Breslow and Day (1980) for individually matched case–control studies, for the estimation of odds ratios (OR) and corresponding 95% confidence intervals (95% CI), adjusting for confounding variables where necessary. Population attributable risk (PAR, expressed as per cent) was computed for any benign thyroid disease using the method described by Breslow and Day (1980). This measure provides an estimate of the incidence of thyroid cancer in a population that can be attributable to a specific risk factor, assuming that the cases represent all the thyroid cancer patients in the population. Stepwise conditional logistic regression analysis (including all the dietary items and adjusting for education) was conducted to identify the most important dietary item(s) independently associated with thyroid cancer. The 95% confidence intervals shown are based on the likelihood ratio test procedure; and the *P*-values for the trend in odds ratios are based on a χ^2^ test for trend. All data management and analyses were conducted using the SPSS and STATA statistical programmes.

## RESULTS

The distribution of 313 thyroid cancer patients (238 females, 75 males) according to selected variables, is shown in Table 1

**Table 1:**
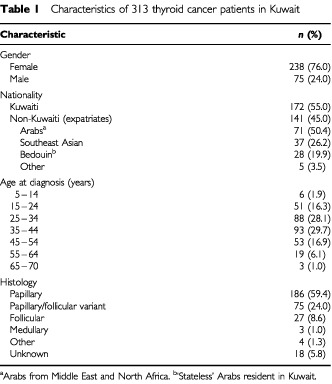
Characteristics of 313 thyroid cancer patients in Kuwait

; 172 (55%) were Kuwaiti nationals, the remainder non-Kuwaitis. Among the latter, the majority (70.3%) were from Arab countries and 26.2% were from Southeast Asia. The majority of cases (74.1%) were diagnosed at a relatively young age (15–44 years). The average age at diagnosis (±s.d.) was 34.7±11 years (range 10–65 years) in women and 39±13.4 years (range 6–69 years) in men; the median age was 35 and 38 years, respectively. There was no difference in the average age at diagnosis between Kuwaiti and non-Kuwaiti patients. Papillary carcinoma (including mixed papillary/follicular variant) was the most common histopathological type accounting for 83.4% of all cases.

### Sociodemographic factors

Table 2

**Table 2:**
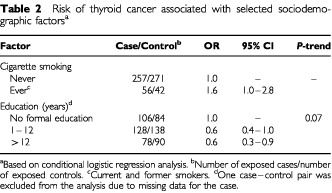
Risk of thyroid cancer associated with selected sociodemographic factors^a^

shows the association between selected sociodemographic factors and thyroid cancer. There was an increased risk of thyroid cancer among individuals who had ever smoked cigarettes (current and former smokers) compared to those who had never smoked (OR=1.6; 95% CI: 1.0–2.8). Individuals with 12+ years of education (professional diploma/university degree) were at a significantly reduced risk compared to those who had no formal education (illiterate/read and write) (OR=0.6; 95% CI: 0.3–0.9).

### Autoimmune and chronic disease

Patients with thyroid cancer had relatively high prevalence of asthma and gall-bladder disease compared to controls (16.6 *vs* 12.8% and 7.7 *vs* 5.8%, respectively) (Table 3

**Table 3:**
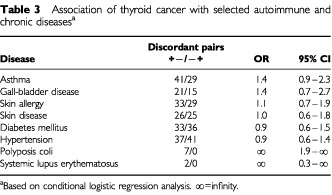
Association of thyroid cancer with selected autoimmune and chronic diseases^a^

). The ORs for other conditions such as skin allergy/disease, diabetes, and hypertension were close to unity. Among other conditions, history of polyposis coli was reported by seven (lower 95% CI: 1.9) and systemic lupus erythematosus by two cases.

### Benign thyroid disease

We examined hyperplastic thyroid diseases (i.e., nodule/adenoma and goitre) only if they had occurred ⩾3 years before the cancer diagnosis (cases)/pseudo-diagnosis (controls) date (Table 4

**Table 4:**
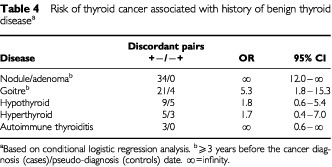
Risk of thyroid cancer associated with history of benign thyroid disease^a^

). There was a strong association with previous hyperplastic thyroid disease. History of thyroid nodule was reported only by cases (*n*=34; 10.9%; lower 95% CI: 12.0). Subjects with a history of thyroid goitre were at about five times increased risk of developing thyroid cancer (OR=5.3; 95% CI: 1.8–15.3). There was no significant increase in risk among subjects with a history of hypothyroidism (OR=1.8) or hyperthyroidism (OR=1.7). Women who had experienced episodes of post-partum thyroiditis were at a significantly increased risk of thyroid cancer (OR=10.2; 95% CI: 2.3–44.8). This is perhaps the first indication in the literature of a possible link between history of post-partum thyroiditis and thyroid cancer. The finding has been discussed in a separate paper (Memon *et al*, 2002). Overall, individuals with a history of any benign thyroid disease were at a considerably high risk of developing thyroid cancer (OR=6.4; 95% CI: 3.4–12.0). The PAR for any benign thyroid disease was about 26% (95% CI: 21.1–30.9).

### Dietary factors

Table 5

**Table 5:**
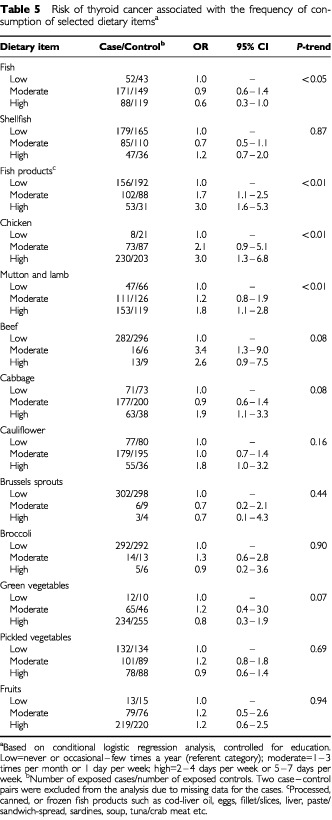
Risk of thyroid cancer associated with the frequency of consumption of selected dietary items^a^

shows the association between frequency of consumption (i.e., low, moderate, high) of selected dietary items and the risk of thyroid cancer. High consumption of fresh fish seemed to have a protective effect (OR=0.6; 95% CI: 0.3–1.0; *P*-trend <0.05). In contrast, there was a strong positive association with high consumption of processed, canned, or frozen fish products (OR=3.0; 95% CI: 1.6–5.3; *P*-trend <0.01). Among other types of meat, high consumption of chicken (OR=3.0; 95% CI: 1.3–6.8; *P*-trend <0.01) as well as mutton and lamb (OR=1.8; 95% CI: 1.1–2.8; *P*-trend <0.01) showed a positive association with thyroid cancer. A significant dose-response effect was observed for all these dietary items. There also seemed to be an increase in risk with consumption of beef, but it was based on a small number of subjects in the moderate and high categories. Considering the four types of cruciferous vegetables included in the dietary section, broccoli and Brussels sprouts were consumed only by a few subjects. There was an increase in risk of thyroid cancer among subjects who reported high consumption of cabbage (OR=1.9; 95% CI: 1.1–3.3) and cauliflower (OR=1.8; 95% CI: 1.0–3.2); but there was no dose-response effect. The ORs for the various categories of consumption of green and pickled vegetables and fruits were close to unity. In the stepwise conditional logistic regression analysis, including all the dietary items and adjusting for education, high consumption of processed fish products (OR=2.2; 95% CI: 1.6–3.0) fresh fish (OR=0.5; 95% CI: 0.4–0.7) and chicken (OR=1.7; 95% CI: 1.2–2.3) were found to be independently associated with the risk of thyroid cancer. We found that among the 53 thyroid cancer patients who reported high consumption of fish products, a large majority also reported a high consumption of fresh fish (98.1%; 52 out of 53) shellfish (67.9%; 36 out of 53) and pickled vegetables (37.7%; 20 out of 53).

## DISCUSSION

To our knowledge, this is the first epidemiological study to examine the major aetiological hypotheses for thyroid cancer in a Middle Eastern population. An international collaborative group has recently published a series of overviews by pooling data from all the case–control studies of thyroid cancer published during the period 1980–1997 (*n*=12) and two unpublished studies (Franceschi *et al*, 1999; Negri *et al*, 1999; Bosetti *et al*, 2001). The majority of these studies, however, have been conducted among the European (*n*=8) and USA (*n*=4) populations; and only two studies have been reported from Asia (China and Japan). Concerning the history of benign thyroid disease and risk of thyroid cancer, the majority of studies have demonstrated a strong link between hyperplastic thyroid disease and thyroid cancer (McTiernan *et al*, 1984; Preston-Martin *et al*, 1987, 1993; Ron *et al*, 1987; Franceschi *et al*, 1989; Kolonel *et al*, 1990; Levi *et al*, 1991; D'Avanzo *et al*, 1995; Mack *et al*, 1999); whereas, for hypo- and hyperthyroidism the majority of studies have shown no material association with thyroid cancer. In most case–control studies, the risk estimates have varied between 10–40 for benign thyroid nodule and 3–8 for goitre. In a number of studies, the risk estimate for thyroid nodule was infinity as the history of the condition was reported only by cases. In a pooled analysis, history of benign thyroid nodule (OR=29.9; 95% CI: 14.5–62.0) and goitre (OR=5.9; 95% CI: 4.2–8.1) was strongly associated with thyroid cancer (Franceschi *et al*, 1999). In agreement with these studies, our study also showed that history of benign thyroid nodule (34 cases *vs* 0 controls) and goitre (OR=5.3) is an important determinant of thyroid cancer. The PAR of 26% for any benign thyroid disease was comparable to that reported in studies from USA (Ron *et al*, 1987) and northern Italy (D'Avanzo *et al*, 1995).

Iodine has an essential role in the production and regulation of thyroid hormones, and both iodine deficiency and excess have been considered as possible risk factors for goitre as well as thyroid cancer (Ward and Ohshima, 1986; Franceschi *et al*, 1990). Chronic hypersecretion of thyroid stimulating hormone (TSH) leads to thyroid follicular cell hyperplasia and hypertrophy (i.e., goitre) and is thought to increase the risk of neoplastic transformation (Williams, 1990). Sub-optimal levels of iodine lead to reduced production of thyroid hormones and this evokes a feedback mechanism to enhance TSH secretion. Conversely, long-term intakes of excessive amounts of iodine can also affect the functioning of thyroid gland and this can lead to elevated TSH levels. It has also been suggested that deficient intakes increase the risk of follicular type and excessive intakes increase the risk of papillary thyroid cancer (World Cancer Research Fund, 1997). Fish, particularly salt-water fish and shellfish, is the best natural source of iodine; whereas, in most diets, bread, dairy products, poultry, and iodised table salt are the main sources of this mineral.

Kuwait, with a population of about 2.2 million, is located along the northwestern borders of the Gulf. Due to its coastal location, fish has always been an important component of the local diet. Currently, about 6000–8000 tons of fish and 2000 tons of shrimp is caught in the territorial waters annually and a similar quantity is imported, mainly from neighbouring Gulf countries. In addition, a large variety of processed, canned, and frozen fish products (including cod-liver oil, eggs, fillet/slices, liver, paste/sandwich-spread, sardines, soup, tuna/crab meat) are imported from different countries. Iodised salt was first introduced in the country in the 1980s and is widely used.

In our study, the stepwise regression analysis showed that high consumption of processed fish products had a positive association with thyroid cancer; whereas, consumption of fresh fish had a protective effect. There was also a significant dose-response relationship with the frequency of consumption of these dietary items. The thyroid cancer patients who reported high consumption of fish products, also reported high consumption of fresh fish (98%), shellfish (68%), and pickled vegetables (38%). It can be inferred that these individuals therefore, had the highest exposure to various types of seafood; and they were also at about two times increased risk of thyroid cancer. The apparently anomalous finding between fresh and processed fish consumption is difficult to explain. The latter may have a much higher concentration of iodine. It is also conceivable that the processing procedure or additives may affect iodine absorption and uptake by the thyroid. Factors other than the iodine content may also be responsible for the increased cancer risk associated with processed fish products. In a review of the epidemiological and experimental data on nutrition and thyroid cancer, a panel of experts concluded that diets with an excessive intake of iodine possibly increase the risk of thyroid cancer (World Cancer Research Fund, 1997). As the dietary assessment in our study was restricted to frequency of consumption of a selected number of indicator food items, reliable measurements of specific nutrients (e.g., iodine content of fish or fish products) were not possible. Therefore, only a general inference on dietary patterns can be justified. Similar findings of an increased risk with habitual high consumption of a variety of seafood have also been reported in a study from Norway (Glattre *et al*, 1993).

A pooled analysis of case–control studies of thyroid cancer, examining fish and shellfish consumption (Bosetti *et al*, 2001), showed that there was no association between high fish consumption and the risk of thyroid cancer (OR=0.88; 95% CI: 0.71–1.10). Among individual studies, a positive association with fish consumption has been reported from Norway (Glattre *et al*, 1993) USA (Ron *et al*, 1987) and China (Preston-Martin *et al*, 1993); no association was found in two studies from northern Europe (Hallquist *et al*, 1994; Galanti *et al*, 1997) Hawaii (Kolonel *et al*, 1990) and Japan (Takezaki *et al*, 1996); whereas, a protective effect was observed in studies from Italy (Franceschi *et al*, 1989) and Sweden (Wingren *et al*, 1993) (Table 6

**Table 6:**
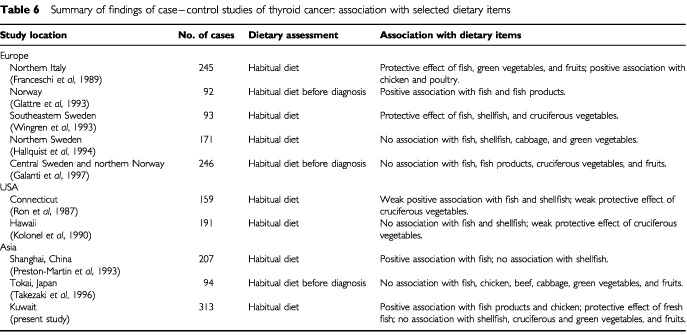
Summary of findings of case–control studies of thyroid cancer: association with selected dietary items

).

Relatively few studies have examined association between consumption of meat, poultry, and dairy products and thyroid cancer. Chicken is the most popular type of meat consumed in Kuwait; and the bulk of the chicken consumed is raised in the local poultry farms. The poultry meat production in the country, for example, increased from about 20 000 tons in 1993 to 39 000 tons in 1999 (Annual Statistical Abstract, 2000). It is well known that crushed oyster shells, fish meal, and iodised salt are essential components of most rations used in commercial poultry farms. In our study, high consumption of chicken was significantly associated with an increased risk of thyroid cancer. There was also a significant dose-response relationship with the frequency of consumption. An increased risk of similar magnitude, for high consumption of chicken and poultry, was reported in a study from Italy (RR=1.98) (Franceschi *et al*, 1989); whereas, no association was found in studies from USA (Ron *et al*, 1987) and Japan (Takezaki *et al*, 1996) (Table 6).

Vegetable and fruit consumption is generally accepted to have a protective effect on a wide range of cancers. For thyroid cancer, studies of vegetable consumption have tended to focus on cruciferous vegetables because they contain thioglucosides, which may be degraded to form goitrogens. However, these vegetables also contain indole components, phenols, and other compounds that may inhibit the development of certain cancers. Among these vegetables, high consumption of cabbage has been implicated in the aetiology of endemic goitre in central Europe, Finland, and some Mediterranean islands (Franceschi *et al*, 1993). In Hawaii, surveys of diet have shown that cabbage and turnips are consumed more frequently by the ethnic groups at the highest risk of thyroid cancer (Goodman *et al*, 1988). The majority of case–control studies, however, have shown a decreased risk of thyroid cancer with increased consumption of cruciferous vegetables, as well as with green vegetables and/or fruits (Ron *et al*, 1987; Franceschi *et al*, 1989; Kolonel *et al*, 1990; Wingren *et al*, 1993) (Table 6). In our study, there was no clear association between consumption of cruciferous vegetables and thyroid cancer. In a review of the data on nutrition and thyroid cancer, a panel of experts concluded that higher intakes of cruciferous vegetables do not increase the risk of thyroid cancer; and diets high in vegetables and fruits possibly decrease the risk of thyroid cancer (World Cancer Research Fund, 1997).

In summary, our study of a Middle Eastern population with relatively high incidence of thyroid cancer (and possibly benign thyroid disease) indicates that about 26% of cases of thyroid cancer may be attributed to previous benign thyroid disease. Our results indicate that high consumption of various types of seafood may also be relevant to the aetiology of thyroid cancer, while an association with chicken consumption is a relatively new finding and needs to be evaluated further.

## References

[bib1] Annual Statistical Abstract2000Ministry of Planning, Kuwait

[bib2] BosettiCKolonelLNegriERonEFranceschiSDal MasoLGalantiMRMarkSDPreston-MartinSMcTiernanALandCJinFWingrenGHallquistAGlattreELundELeviFLinosDLa VecchiC2001 A pooled analysis of case–control studies of thyroid cancer. VI. Fish and shellfish consumption Cancer Causes Control 12 375382 11456234 10.1023/a:1011267123398

[bib3] BreslowNEDayNE1980 The analysis of case–control studiesInStatistical Methods in Cancer Research Vol. IIARC Scientific Publications No. 32Lyon: International Agency for Research on Cancer 7216345

[bib4] D'AvanzoBLa VecchiaCFranceschiSNegriETalaminiR1995 History of thyroid diseases and subsequent thyroid cancer risk Cancer Epidemiol Biomarkers Prev 4 193199 7606193

[bib5] FranceschiSFassinaATalaminiRMazzoliniAVianelloSBidoliESerrainoDLa VecchiaC1989 Risk factors for thyroid cancer in northern Italy Int J Epidemiol 18 578584 2807659 10.1093/ije/18.3.578

[bib6] FranceschiSTalaminiRFassinaABidoliE1990 Diet and epithelial cancer of the thyroid gland Tumori 76 331338 2205027 10.1177/030089169007600406

[bib7] FranceschiSBoylePMaisonneuvePLa VecchiaCBurtADKerrDJMacFarlaneGJ1993 The epidemiology of thyroid carcinoma Crit Rev Oncog 4 2552 8416150

[bib8] FranceschiSPreston-MartinSDal MasoLNegriELa VecchiaCMackWJMcTiernanAKolonelLMarkSDMabuchiKJinFWingrenGGalantiRHallquistAGlattreELundELeviFLinosDRonE1999 A pooled analysis of case–control studies of thyroid cancer. IV. Benign thyroid diseases Cancer Causes Control 10 583595 10616827 10.1023/a:1008907227706

[bib9] GalantiMRHanssonLBergströmRWolkAHjartåkerALundEGrimeliusLEkbomA1997 Diet and the risk of papillary and follicular thyroid carcinoma: a population-based case–control study in Sweden and Norway Cancer Causes Control 8 205214 9134245 10.1023/a:1018424430711

[bib10] GlattreEHaldorsenTBergJPStensvoldISolvollK1993 Norwegian case–control study testing the hypothesis that seafood increases the risk of thyroid cancer Cancer Causes Control 4 1116 8431525 10.1007/BF00051708

[bib11] GoodmanMTYoshizawaCNKolonelLN1988 Descriptive epidemiology of thyroid cancer in Hawaii Cancer 61 12721281 3342383 10.1002/1097-0142(19880315)61:6<1272::aid-cncr2820610636>3.0.co;2-8

[bib12] HallquistAHardellLDegermanABoquistL1994 Thyroid cancer: reproductive factors, previous diseases, drug intake, family history and diet. A case–control study Eur J Cancer Prev 3 481488 7858480

[bib13] KolonelLNHankinJHWilkensLRFukunagaFHHindsMW1990 An epidemiologic study of thyroid cancer in Hawaii Cancer Causes Control 1 223234 2102295 10.1007/BF00117474

[bib14] LeviFFranceschiSLa VecchiaCNegriEGulieCDuruzGScazzigaB1991 Previous thyroid disease and risk of thyroid cancer in Switzerland Eur J Cancer 27 8588 1826448 10.1016/0277-5379(91)90069-p

[bib15] MackWJPreston-MartinSBrensteinLQianDXiangM1999 Reproductive and hormonal risk factors for thyroid cancer in Los Angeles County females Cancer Epidemiol Biomarkers Prev 8 991997 10566554

[bib16] McTiernanAMWeissNSDalingJR1984 Incidence of thyroid cancer in women in relation to previous exposure to radiation therapy and history of thyroid disease J Natl Cancer Inst 73 575581 6590909

[bib17] MemonADarifMAl-SalehKSureshA2002 Epidemiology of reproductive and hormonal factors in thyroid cancer: evidence from a case–control study in the Middle East Int J Cancer 97 8289 11774247 10.1002/ijc.1573

[bib18] NegriERonEFranceschiSDal MasoLMarkSDPreston-MartinSMcTiernanAKolonelLKleinermanRLandCJinFWingrenGGalantiMRHallquistAGlattreELundELeviFLinosDBragaCLa VecchiaC1999 A pooled analysis of case–control studies of thyroid cancer. I. Methods Cancer Causes Control 10 131142 10231162 10.1023/a:1008851613024

[bib19] ParkinDMWhelanSLFerlayJRaymondLYoungJ1997 Cancer incidence in five continents Vol. VII IARC Sci. Publ. No. 143Lyon: IARC

[bib20] Preston-MartinSBernsteinLPikeMCMaldonadoAAHendersonBE1987 Thyroid cancer among young women related to prior thyroid disease and pregnancy history Br J Cancer 55 191195 3814488 10.1038/bjc.1987.36 PMC2002082

[bib21] Preston-MartinSJinFDudaMJMackWJ1993 A case–control study of thyroid cancer in women under age 55 in Shanghai (People's Republic of China) Cancer Causes Control 4 431440 8218875 10.1007/BF00050862

[bib22] RonEKleinermanRABoiceJDLiVolsiVAFlanneryJTFraumeniJrJF1987 A population-based case–control study of thyroid cancer J Natl Cancer Inst 79 112 3474436

[bib23] RonELubinJHShoreREMabuchiKModanBPotternLMSchneiderABTuckerMABoiceJrJD1995 Thyroid cancer after exposure to external radiation: a pooled analysis of seven studies Radiat Res 141 259277 7871153

[bib24] RonE1996 Epidemiology of thyroid cancerInCancer Epidemiology and PreventionSchottenfeld D, Fraumeni Jr JR (eds)pp10001021Oxford: Oxford University Press

[bib25] TakezakiTHiroseKInoueMHamajimaNKuroishiTNakamuraSKoshikawaTMatsuuraHTajimaK1996 Risk factors of thyroid cancer among women in Tokai, Japan J Epidemiol 6 140147 8952218 10.2188/jea.6.140

[bib26] WardJMOhshimaM1986 The role of iodine in carcinogenesis Adv Exp Med Biol 206 529542 3591538 10.1007/978-1-4613-1835-4_37

[bib27] WilliamsED1990 TSH and thyroid cancer Horm Metab Res Suppl 23 7275 2210635

[bib28] WingrenGHatschekTAxelsonO1993 Determinants of papillary cancer of the thyroid Am J Epidemiol 138 482491 8213752 10.1093/oxfordjournals.aje.a116882

[bib29] World Cancer Research Fund in association with American Institute for Cancer Research1997 Food, Nutrition and the Prevention of Cancer: A Global PerspectiveWashington, DC: American Institute for Cancer Research 10.1016/s0899-9007(99)00021-0 10378216

